# A Chemical-Pressure-Induced Phase Transition Controlled by Lone Electron Pair Activity

**DOI:** 10.1021/acs.jpclett.2c02582

**Published:** 2022-10-17

**Authors:** Eduardo O. Gomes, Amanda F. Gouveia, Lourdes Gracia, Álvaro Lobato, J. Manuel Recio, Juan Andrés

**Affiliations:** † Departament de Química Física i Analítica, Universitat Jaume I, 12071 Castelló de la Plana, Spain; ‡ MALTA-Consolider Team and Departamento de Química Física, Universidad Complutense de Madrid, 28040 Madrid, Spain; ¶ MALTA-Consolider Team and Departamento de Química Física y Analítica, Universidad de Oviedo, 33006 Oviedo, Spain; § MALTA-Consolider Team and Departament de Química Física i Analítica, Universitat Jaume I, 12071 Castelló de la Plana, Spain; ∥ MALTA-Consolider Team and Department of Physical Chemistry, University of Valencia (UV), 46100 Burjassot, Spain

## Abstract

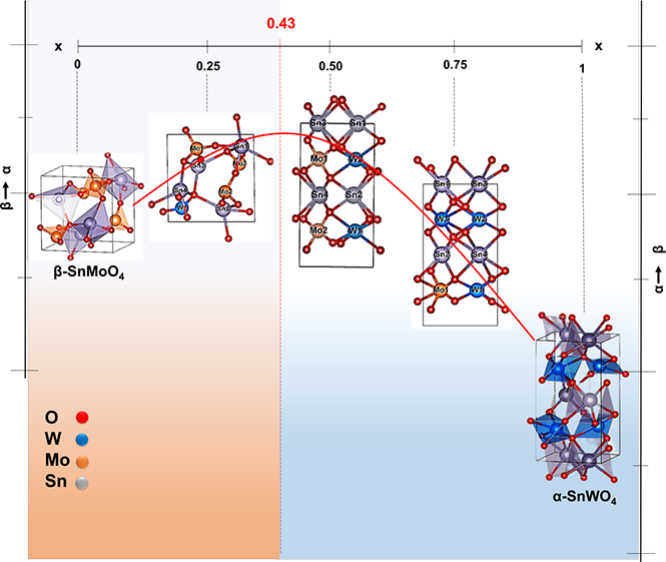

The chemical pressure approach offers a new paradigm for property control in functional materials. In this work, we disclose a correlation between the β → α pressure-induced phase transition in SnMoO_4_ and the substitution process of Mo^6+^ by W^6+^ in SnMo_1–*x*_W_*x*_O_4_ solid solutions (*x* = 0–1). Special attention is paid to discriminating the role of the lone pair Sn^2+^ cation from the structural distortive effect along the Mo/W substitution process, which is crucial to disentangle the driven force of the transition phase. Furthermore, the reverse α → β transition observed at high temperature in SnWO_4_ is rationalized on the same basis as a negative pressure effect associated with a decreasing of W^6+^ percentage in the solid solution. This work opens a versatile chemical approach in which the types of interactions along the formation of solid solutions are clearly differentiated and can also be used to tune their properties, providing opportunities for the development of new materials.

Chemical pressure is an efficient tool to tune properties and create functional materials.^1−6^ The intentional introduction or substitution of chemical elements into a material is a fundamental process to modify its intrinsic structure and properties while changing its composition.^7−10^ Usually, chemical pressure is mainly associated with structural effects provoked by the different sizes of the doping agents with respect to the host structure. These strains can recreate the response of the material under stress conditions facilitating the equivalence between physical or mechanical pressure and chemical pressure. The concomitant changes in the bonding network introduced by the guest elements have been less studied. They also produce an impact on the behavior of the materials^5,9,11^ that are difficult to separate from the pure structural perturbations. The knowledge of these new interactions is crucial for precise control of the properties emerging through these chemical doping processes.

Solid solutions of semiconductors are pertinent examples to illustrate how the interactions associated with the doping process mimic the effect of thermodynamic variables (pressure and temperature) and also modulate the properties of advanced materials with extended functionalities (see for example refs (5, 12)). Among these solid solutions, the ternary metal tungstate- and molybdate-based compounds form an important class of multifunctional materials with a combination of covalent, ionic, and metallic bonding that has been widely studied due to their fundamental and industrial interest.^13−15^ In spite of being in the next period, tungsten shows a similar atomic radius as molybdenum due to the well-known lanthanide contraction. This good size matching guarantees the alloying of the two semiconductors in a wide composition range and avoids the phase separation that otherwise would occur originated by large lattice-mismatching strains.

In the case of SnMo_1–*x*_W_*x*_O_4_ (*x* = 0–1), the two end compounds show however different space groups at room conditions: SnMoO_4_ crystallizes in the *P*2_1_3 cubic β phase, whereas the *Pnna α* phase of SnWO_4_ is orthorhombic. The band structure is different too. At room conditions, α-SnWO_4_ presents a small and indirect band gap,^16^ while β-SnMoO_4_ presents a band gap around 1.5 eV higher.^17^ As announced by Walsh et al.,^18^ compounds combining lone pair *ns*^2^ cations (Sn^2+^, Pb^2+^, ···) and *d*^0^ transition metals (Mo^6+^, W^6+^, ···) constitute promising candidates to improve photocatalyst properties in hydrogen economy thanks to the possibilities they offer to tune their electronic band structure.^17^ Moreover, the polyhedral distortions of both cation environments lead to metallic off-center positions well-studied for the potential use of these compounds as multiferroic materials.^19^

A transition between the two structures should be expected at some intermediate *x* composition. Moreover, around 0.8 GPa, β-SnMoO_4_ transforms to the α phase, whereas α-SnWO_4_ is observed to transform to the β phase at high temperature.^20^ Therefore, by increasing (decreasing) the W content, the actual symmetry of the system should change as happens with increasing pressure (temperature) in the pure β-SnMoO_4_ (α-SnWO_4_) compound. All these possibilites are illustrated in Figure 1.

**Figure 1:**
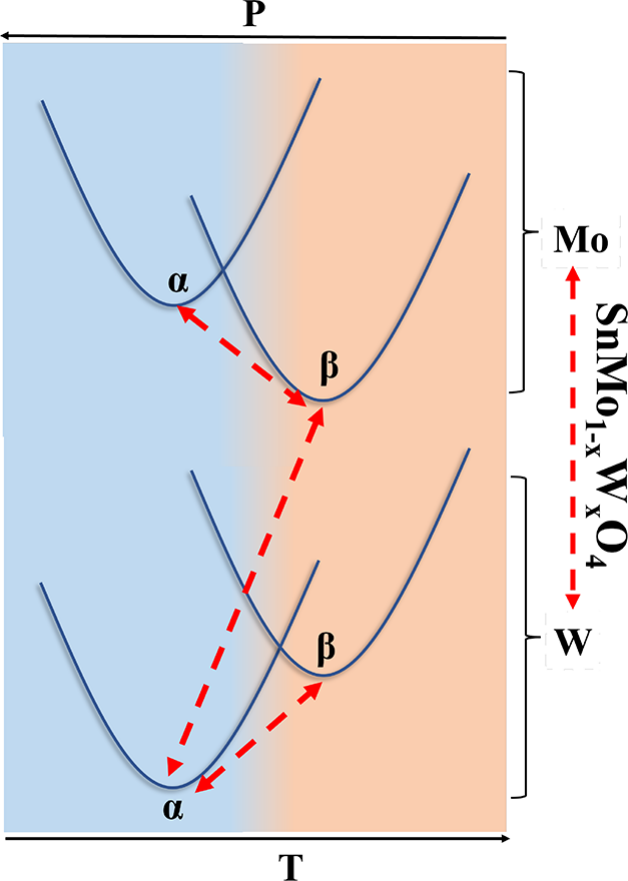
Expected energy-volume scenario highlighting potential pressure-, composition-, and temperature-induced transitions in the SnMo_1–*x*_W_*x*_O_4_ solid solution. Arrows from top to bottom indicate phases connected across these transitions.

Given the size matching between both *d*-type elements, the factors associated with differences in the electronic structure of the two transition metals should be key to understand the sought-after analogy between physical and chemical pressure. Fortunately, a deep understanding of the structural distortions of *d*^0^ cations, as Mo^6+^ and W^6+^, in crystalline environments involving lone pair cations (Sn^2+^) was provided by Halasyamani et al.^19,21^ These authors remark the differentiating role of the atomic electronegativity to classify Mo^6+^ as a strong distorter and W^6+^ as a moderate distorter.^21^

In the present paper, we address the doping process along the SnMo_1–*x*_W_*x*_O_4_ solid solution (*x* = 0–1). The structural and electronic interplay associated with the W^6+^/Mo^6+^ exchange is investigated to show how the properties of the solid solution can be modulated by the precise control of its composition. It is an attractive example to highlight that the new interactions emerging from the doping process are responsible for the chemical pressure impact on the material. Our challenge is twofold. First, we seek to illustrate the equivalence between the thermodynamic variables and the chemical pressure effects so we can substitute the impact of pressure and/or temperature with chemical doping. Second, we pursue to make explicit that this equivalence has not a genuine structural origin but is caused by the different chemistry of both *d*^0^ cations when coupled to the lone pair Sn^2+^ cation. Relying on the work of Halasyamani and collaborators^19,21^ and the revised classical lone pair model of Walsh et al.,^18^ a coherent explanation of the different behavior of both transition metal (T) compounds can support our findings.

To accomplish these goals, we carry out an extensive structural optimization of α and β crystalline structures in SnMo_1–*x*_W_*x*_O_4_ solid solutions with *x* = 0, 0.25, 0.50, 0.75, and 1. To clearly understand how chemical doping affects globally and locally the structures, we first briefly describe the unit cells of the β and α phases. The β phase structure belongs to the cubic *P*2_1_3 space group. This structure is constituted by slightly deformed [TO_4_] tetrahedra (T = Mo, W), which are interconnected with strongly distorted [SnO_6_] octahedra. The α phase structure belongs to the orthorhombic *Pnna* space group. It is composed by layers of [TO_4_] tetrahedra (T = Mo, W) stacked along the *c* direction and separated by layers of Sn^2+^ cations, which are eightfold coordinated by oxygen atoms, forming [SnO_8_] cubic antiprism polyhedra (see Figure 2).

**Figure 2:**
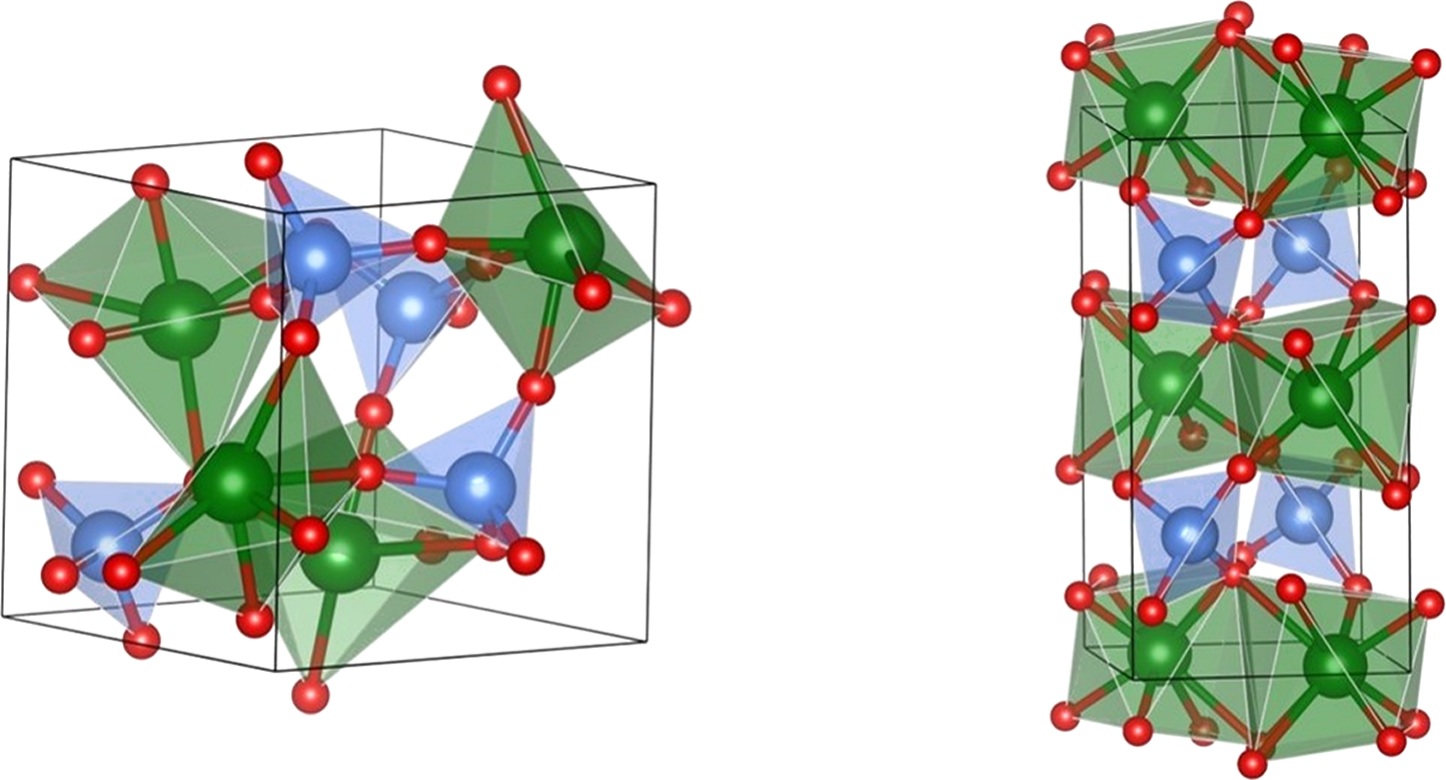
Polyhedral view of the β (left) and α (right) unit cells. SnO_6_ (β) and SnO_8_ (α) polyhedra are shown in green, whereas [TO_4_] tetrahedra are depicted in blue.

The calculated static lattice parameters and optimized atomic positions for the Mo and W compounds in both α and β polymorphs are collected in the Supporting Information (SI) file (see Tables S2 and S3, the accuracy in the lattice parameters is within 10^–3^ Å). The cubic cell parameters are 7.131 and 7.073 Å, for β-SnMoO_4_ and β-SnWO_4_, respectively. Lattice parameters of the α-SnMoO_4_ are *a* = 5.597 Å, *b* = 10.717 Å, and *c* = 5.423 Å, while for α-SnWO_4_, they are *a* = 5.605 Å, *b* = 10.574 Å, and *c* = 5.498 Å. The similarity between these values in both compounds evidences that differences in the T^6+^ atomic size do not introduce meaningful changes in the lattice parameters. However, looking at the local coordination polyhedra, we find that MoO_4_ distortions calculated using the quadratic elongation and bond angle variance parameters^22^ are slightly larger than in WO_4_ tetrahedra, as anticipated by Halasyamani in the analysis of second order Jahn-Teller effects in *d*^0^ T cations. We also notice that transition metal polyhedral distortions are greater in the α than in the β phase, which have implications in the phase stability as we will discuss later. Distortion parameters are collected in Table S4 of the SI file.

The available experimental observations found that the β phase is thermodynamically favored at room conditions for SnMoO_4_ and at high temperature for the SnWO_4_ compound. As pointed out above, pressure induces a β → α phase transition in the SnMoO_4_, whereas α-SnWO_4_ transforms to the β phase at high temperature.^20^ Our simulations successfully reproduce the experimental facts and quantify the pressure of the β-SnMoO_4_ → α-SnMoO_4_ transition at 0.77 GPa and the α-SnWO_4_ → β-SnWO_4_ transition temperature at 1200 K (see Figure S1). Figure 3 shows an schematic representation of the phase diagram of both compounds.

**Figure 3:**
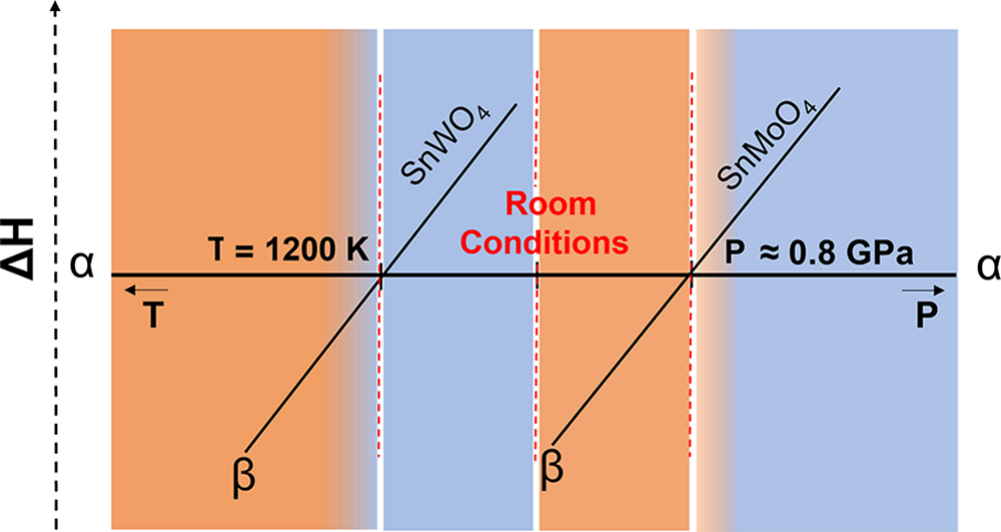
Pressure and temperature regions of α and β stability for both SnMoO_4_ and SnWO_4_ compounds.

We can alternatively move from the β phase to the α one, just replacing Mo by W atoms or from the α phase to the β one replacing W by Mo atoms. In what follows, we demonstrate that these solid–solid transitions can be controlled through the new interactions emerging in chemical doping processes. Being pressure and temperature opposite effects, it is interesting to resort to the concept of chemical pressure to provide a unified insight of the whole phenomena.

For the calculations of the SnMo_1–*x*_W_*x*_O_4_ solid solutions, the substitution of Mo by W atoms was performed with molar fractions of *x* = 0, 0.25, 0.50, 0.75, and 1, both for the α and β phases. Further details of the simulation procedure are given in the SI file along with the optimized structure parameters and atomic positions of these solid solutions (see Tables S2 and S3). The comparison of the calculated cell parameters of these solid solutions with other available experimental and theoretical values shows a good agreement.^23^

The energy evolution of the SnMo_1–*x*_W_*x*_O_4_ solid solution is as follows. Up to *x* = 0.43, the stable structure corresponds to the cubic β phase with an energy lower than the α one with the same composition. From *x* = 0.43 to 1, the orthorhombic structure of the α phase becomes the stable one, since it has a lower energy than the corresponding composition of the β phase. Therefore, our simulations are able to identify a structural phase transition from the β to the α phase when the doping of SnMoO_4_ with W atoms amounts to *x*_W_ = 0.43. Likewise, starting with SnWO_4_ and substituting W by Mo, the α → β transition is predicted at *x*_Mo_ = 0.57. It is clear from these results that by selecting the precise amount of the doping agent we can control the actual structure of the solid solution mimicking the effect of pressure and temperature. We quantify the pressure exerted by W atoms in the *x*_W_ = 0.43 solid solution as 0.77 GPa, since this value is the physical pressure found for the β → α transition in SnMoO_4_. Likewise, the temperature of 1200 K determined for the α → β transition in SnWO_4_ is associated with *x*_Mo_ = 0.57 (see Figure S1). Under the unified perspective provided by chemical pressure, we see that both pressure and temperature effects on these pure SnMoO_4_ and SnWO_4_ compounds can be reproduced.

Overall, we can conclude that chemical pressure provides an efficient way to compress and expand crystalline structures. As compressions are commonly related to positive pressures, the equivalence between physical and chemical pressure is easily understood. Concerning lattice expansions, the same formal ideas can be used to link the effect of chemical pressure with negative physical pressures. A general correlation between physical (*p* in GPa) and chemical (*x* in molar fraction) pressure is presented in Figure 4. In each of the two pure compounds, we have associated *x* values with the physical pressures that show the same Δ*G*_β–α_ values. For instance, the value of Δ*G*_β–α_ = 0.218 eV for *x*_W_ = 0.50 corresponds to a physical pressure of −2.2 GPa when applied to the SnWO_4_ and to 1.7 GPa when exerted on SnMoO_4_. Using this correspondence, in the SnMo_1–*x*_W_*x*_O_4_ solid solution with a molar fraction of *x*_W_ = 0.43, a value of −3 GPa is obtained for the pressure at which the transition from the α to the β phase takes place.

**Figure 4:**
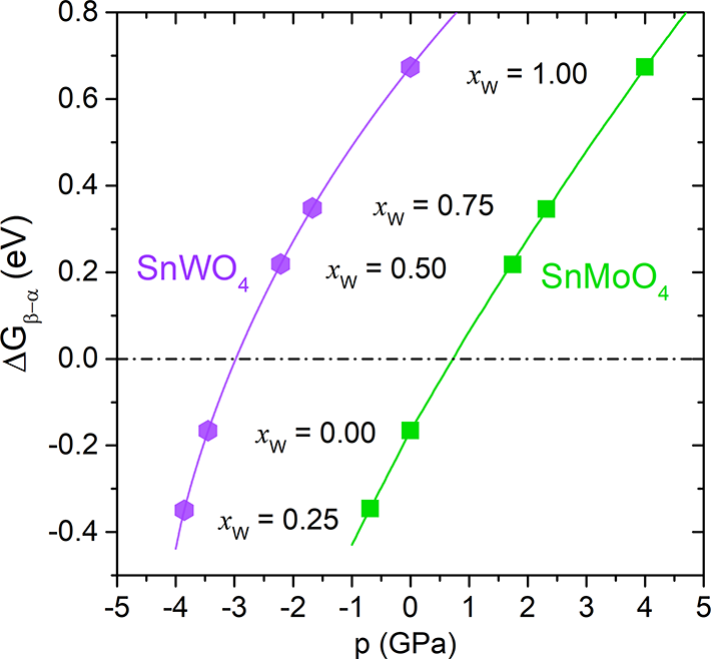
Chemical pressure-physical pressure correspondence for the SnWO_4_ (purple) and SnMoO_4_ (green) pure compounds.

The possibility of achieving negative pressure regions by chemical doping has not to be neglected as recently remarked by Wang et al.:^4^ “*It is a big challenge but of great value to pursue a path to gain a large negative pressure and modulate the properties in solid state matter*”. In our case, we have estimated that SnMo_1–*x*_W_*x*_O_4_ solid solution with *x* = 0.50 and *x* = 0.75 reproduce negative pressures of −2.2 and −1.7 GPa in the α-SnWO_4_ compound, respectively. Our calculated band gaps of these solid solutions are 1.38 and 1.61 eV, respectively, being the band gap in the α-SnWO_4_ pure compound 2.14 eV (see Tables S1 and S2). Our results thus provide an example of how negative pressures through chemical doping can modulate the band gap in these materials. Indeed, new synthetic methods like heterostructural alloying might be used to stabilize these negative pressure polymorphs.^24^

The question that remains is which is the driving force of these phase transitions if Mo^6+^ and W^6+^ cations have a similar size. To answer this question, we analyze the role played by the lone electron pair (*E*) of the Sn^2+^ cations. The activity of the lone pair is known to be dependent on the electronegativity of the ligand.^25^ As emphasized by Lin et al.,^5^ chemical pressure induces modifications in the material behavior caused not only by size effects but also due to new chemical interactions emerging from changes in the electronic structure. In agreement with previous studies of Sn oxides containing *d*^0^ cations,^19,21^ the greater degree of distortion in Mo than W polyhedra is clearly illustrated by the specific features of the charge density and the electron localization function. Our ELF analysis reveals the existence of SnO_3_*E* and SnO_4_*E* units within the octahedra and cubic antiprism polyhedra, respectively (see Figure S2). The oxygens of the SnO_3_*E* units correspond to the three shortest Sn–O distances of the SnO_6_ octahedra leading to the (approximated) *AX*_3_*E* trigonal pyramidal geometry of the VSEPR model. Similarly, the SnO_4_*E* units are constituted by the oxygen atoms with the shortest (2 + 2) Sn–O distances in the SnO_8_ cubic antiprism, resembling the *AX*_4_*E* seesaw VSEPR geometry. It is very relevant to notice that SnO_3_*E* units only appear in the β phase, whereas SnO_4_*E* units are associated with the α phase. Both Sn^2+^ and T^6+^ centered coordination polyhedra are linked by bridging oxygens (O_b_). This feature is key to understand the preference for the most dense packing found in the α phase either at normal conditions in SnWO_4_ or at high pressure in SnMoO_4_.

We propose an extension of the revised classical lone pair model of Walsh et al.^18^ that can advance in the final solution. In the extended molecular orbital-type diagram depicted in Figure 5, we illustrate how the interaction between the lone electron pair of tin in the 5*s* orbital and the 2*p* orbital of the oxygen bridging both polyhedra generates an antibonding state (populated first with a loosely pair of electrons) that is able to mix with the empty 5*p* orbital of tin and finally stabilize the two antibonding electrons. The amount of stabilization depends on the degree of interaction of the lone electron pair 5*s* orbital with the 2*p* orbital of the bridging oxygen. As this interaction depends on the relative energy of these orbitals, the electronegativity of the transition metal to which the bridging oxygen is bonded plays a role since it modifies the energy of the 2*p* oxygen orbital.

**Figure 5:**
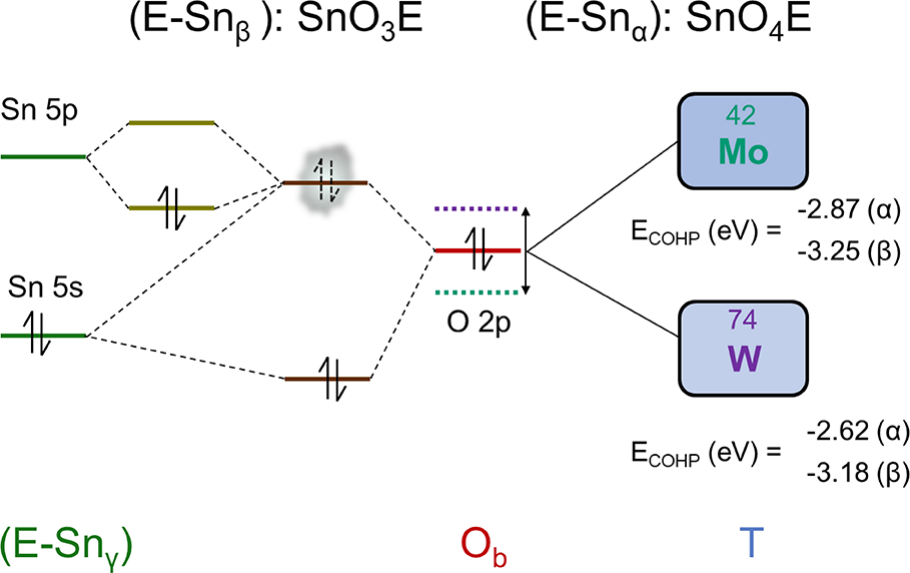
Extended MO energy diagram for the Sn-O_b_-T interaction.

According to Halasyamani,^19^ the strong distorted character of Mo^6+^ is associated with a higher electronegativity when compared with the moderate distorter W^6+^. Such a higher electronegativity of Mo results into a stronger covalent Mo–O_b_ bond leading to an energy lowering of the 2*p* O_b_ orbital. The hybridization between the 5*s* and 5*p* states of the Sn^2+^ cation is then facilitated in a greater degree in the Mo compound, increasing the stereoactivity of the lone electron pair. These conclusions are further supported by the values obtained in the Crystal Orbital Hamilton Population (COHP) analysis^26−28^ of the Sn–O bonds. Systematically, COHP interaction energies are revealed to be stronger for Mo pure phases (−2.865 eV α and −3.249 eV β) than for the W ones (−2.620 eV α and −3.178 eV β). The COHP analysis also evidences the greater lone pair activity of the Sn^2+^ cation in the Mo than in the W compound, since the calculated overlapping Sn–O values are 0.059 (α) and 0.073 (β) in SnMoO_4_ and 0.049 (α) and 0.070 (β) in SnWO_4_.

Once we have linked the presence of the Mo^6+^ cation with a higher stereoactivity of the lone pair cation, we argue whether a higher activity shows a preference for the SnO_3_*E* or SnO_4_*E* units. According to the VSEPR model, it seems reasonable to associate with the more rigid unit (SnO_3_*E*) the existence of greater lone pair effects, since this unit has a lower number of structural degrees of freedom. Notice that the SnO_3_*E* unit is only present in the β phase. The COHP interaction energies collected above also reveal greater values for the β than for the α phase. Therefore, we have found that higher (lower) COHP values in the stable β (α) phase are concomitant with the greater (lower) activity of the lone pair in the Mo (W) compound. Moreover, as pressure increases, the energy costs due to the strained energy in the SnO_3_*E* unit are finally released thanks to the transition to the α phase. In the case of the Mo compound, the energetic balance is favorable due to the increasing in the coordination number. Similarly, the preference for the most rigid and more active lone pair of the β phase in the case of the W compound at negative pressure compensates for the decrease in the coordination number when transforming from the α phase. We assume that these arguments can be transferred to the high-temperature regime, since volumes are the same at the transition temperature and at the transition negative pressure.

In summary, the behavior of the SnMo_1–*x*_W_*x*_O_4_ solid solution facilitates the linking between physical and chemical pressure. We have shown that by replacing Mo^6+^ by W^6+^, the same phase transition as the one induced by physical pressure is observed. We have been able to map one magnitude into the other, thus providing quantitative proof of their equivalence in positive and negative regions. One of our findings has been to associate the preference of the *d*^0^ transition metal with the lone pair activity, i.e., either with the SnO_3_*E* or the SnO_4_*E* units. Such a linking is supported by the structural analysis of Halasyamani et al.^19,21^ and the extended MO diagram of Walsh^18^ in *d*^0^ compounds containing lone electron pairs. Our results are able to explain the preference for the densest packing found in the α phase either at normal conditions when the moderate distorter cation W^6+^ is involved or at high pressure in SnMoO_4_. This study highlights the usually overlooked role played by the electronic structure of the guest atom when discussing chemical pressure effects. Since size effects are not meaningful in our system due to similar ionic radii of the two 3*d* cations, electronic structure modifications are here the main responsible to control the thermodynamic stability of particular compositions of the SnMo_(1–*x*)_W_*x*_O_4_ solid solution by appropriate chemical doping. To the best of our knowledge, this is the first time a phase transition induced by the modification of the lone pair activity upon chemical substitution is reported. We believe that experimental proof of such a transition would constitute a plausible challenge for being detected in the laboratory. Extensions to other crystal families containing *ns*^2^ lone pair cations (i.e., Pb, Se, Bi, etc.) and covering interesting phenomena such as multiferroic behavior, low thermal conductivity, or nonlinear optics are worth to be explored following our approach, since lone pair activity might be used to effectively design new functional materials.

## Computational Methods

First-principles calculations under the density functional theory (DFT) framework employing the functionals B3LYP and HSE06 have been performed (see Table S1). The HSE06 hybrid functional provides an overall better agreement with the experimental results regarding lattice parameters and band gap values and was used in our work unless otherwise specified. The electron localization function (ELF) scalar field^29^ was evaluated to identify core, valence, and electron pairs regions of the optimized structures, whereas the crystal orbital Hamilton population^26−28^ (COHP) analysis was used to calculate bonding interaction energies. Pressure, temperature, and composition stability regions are determined for the two structures of the solid solution evaluating Gibbs energy differences. See further details in the computational section of the Supporting Information file.

## Supporting Information Available

Computational details of the electronic structure calculations of the α and β SnMo_1–*x*_W_*x*_O_4_ solid solutions. Lattice and EOS parameters, band gap values (Tables S1 and S2), and optimized atomic positions (Table S3) for the solid solutions studied. Polyhedral distortion parameters of the MoO_4_ and WO_4_ tetrahedra in the β and α phases (Table S4). Gibbs energy-temperature diagram for the α and β SnWO_4_ phases (Figure S1). ELF isosurfaces of the Sn^2^ + lone electron pairs in the β and α phases (Figure S2) (PDF)
Transparent Peer Review report available (PDF)
