# Supplementary material for: A Chemical-Pressure-Induced Phase Transition Controlled by Lone Electron Pair Activity
Source: J Phys Chem Lett. 2022 Oct 17;13(42):9883–8. doi: 10.1021/acs.jpclett.2c02582 (PMC9619963; doi:10.1021/acs.jpclett.2c02582)
Supplement: Supplementary file 1 — jz2c02582_si_001.pdf [file jz2c02582_si_001.pdf]

A Chemical Pressure-Induced Phase Transition Controlled by Lone Electron Pair Activity

Eduardo O. Gomes[1], Amanda F. Gouveia[1], Lourdes Gracia[1,2], ´Alvaro Lobato*[,3], J. Manuel Recio*[,4],¶ and Juan Andrés[5]

[1]Departament de Química Física i Analítica, Universitat Jaume I, 12071, Castelló de la Plana, Spain

[2]MALTA-Consolider Team and Department of Physical Chemistry, University of Valencia (UV), 46100 Burjassot, Spain.

[3]MALTA-Consolider Team and Departamento de Química Física, Universidad Complutense de Madrid, 28040, Madrid, Spain

[4]MALTA-Consolider Team and Departamento de Química Física y Analítica, Universidad de Oviedo, 33006 Oviedo, Spain

[5]MALTA-Consolider Team and Departament de Química Física i Analítica, Universitat Jaume I, 12071 Castelló de la Plana, Spain

*Corresponding authors: a.lobato@ucm.es and jmrecio@uniovi.es

1.-Computational details

a) General Aspects

The structural, electronic properties and EOS of crystalline $SnMoO_4$ and $SnWO_4$ pure compounds were evaluated under the periodic DFT framework using the *CRYSTAL17* package.[1,2] To study the influence of different approximations for exchange and correlation contributions to the DFT energy, a complete structure optimization by using the HSE06[3,4] and B3LYP[5] functionals has been performed.

The atoms were described using pseudopotential basis sets: tungsten was described by a large-core ECP, derived by Hay and Wadt and modified by Cora et al.,[6] molybdenum by Mo-976-311 (d631)G,[7] tin by Sn_ECP28MDF-411(51d) G,[8] and oxygen by O_6-31d1G (all-electron).[9] The accuracy of the evaluation of the Coulomb and exchange series was controlled by five thresholds, whose adopted values were 10^{-7}(overlap threshold for Coulomb integrals), 10^{-7} (penetration threshold for Coulomb integrals), 10^{-7} (overlap threshold for HF exchange integrals), 10^{-7} and 10^{-14} (pseudo-overlap for HF exchange series), which assure a convergence in total energy better than 10^{-7} Hartree in all cases. The percent of Fock/Kohn–Sham matrices mixing has been set to 40 (IPMIX= 40).

The *CRYSTAL* program can perform an automatic scan over the volume to compute energy *E versus* volume *V* data that are then described by the third-order Birch−Murnaghan (BM-3) EOS.[10,11] For each volume, a full *V*-constrained geometry optimization was performed. As a result, the pressure dependence of the unit cell structure was determined, as well as the volume/pressure dependence of the total energy and enthalpy. In addition, an automatic scheme for computing the quasi-harmonic approximation (QHA) crystal properties has been used, considering a volume range extending from a − 3% compression to a + 6% expansion around the equilibrium unit cell volume. The band structures and projected densities of states (DOS) on atoms and orbitals were performed to analyze the electronic structure.

Electron Localization Function (ELF) and Crystal Orbital Hamilton Population (COHP) analysis were obtained from single point calculations using VASP[12,13] at the optimized unit cell obtained from HSE06 CRYSTAL calculations. In this VASP calculations, we used the Perdew-Burke-Ernzerhof (PBE) exchange correlation functional[14] and *k*-point gamma-centered Monkhorst-Pack meshes with a reciprocal spacing of 2π x 0.1 $Å^{-1}$. A kinetic energy cutoff of 600 eV for the plane wave basis set expansion was used to solve Kohn−Sham equations. The pseudopotentials utilized for Mo, W Sn and O atoms were standard projector-augmented wave pseudopotentials[15] provided in VASP code. The valence electrons considered for each atomic species are Mo ($4s^2$ $4p^6$ $4d^5$ $5s^1$), W ($5s^2$ $5p^6$ $5d^4$ $6s^2$), Sn ($4d^{10}5s^25p^2$) and O ($2s^2$ $2s^4$). COHP and the negative of the COHP integrated to the Fermi level were calculated by using the LOBSTER package.[16,17]. PbeVaspFit2015 atomic basis were used to project the plane waves as implemented in the LOBSTER code. Specifically, the following atomic orbitals for each atomic species were used: O 2s 2p, Sn 4d 4p 4s 5p 5s, W 5d 5p 5s 6s and Mo 4d 4p 4s 5s.

b) Solid Solutions

In both structures (α and β), the number of formula units is Z=4. If one, two or three of the Mo positions are replaced by one, two or three W atoms, a 25%, 50% or 75% of substitution will be obtained. In the case of a 50% replacement, there are 3 different

possibilities concerning the relative positions of the Mo and W atoms in the unit cell. We have computed all of them and selected the most energetically favorable.

The replacement of the Mo by W atoms implies a decrease of the cell parameters, except for *b* parameter which tend to increase for α solid solution. However, when the substitution occurs with the solid solution containing half of the substituted W and Mo atoms in *β* solid solution (*β*-$SnW_{0.50}Mo_{0.50}O_4$), the cubic parameter presents an orthorhombic distortion with values of 7.089, 7.102 and 7.110 Å. The substitution process of W by Mo cations induces variations in the atomic coordinates of the neighbor oxygen anions, indicating the existence of structural and electronic distortions in the local coordination of these cations.

2.- Results

Table S1. Optimized lattice parameters (Å) and band gap energy (E_{gap}, eV) of β-$SnMoO_4$, *β*-$SnWO_4$, *α*-$SnWO_4$ and *α*-$SnMoO_4$ along with the differences (%) with respect to the experimental values.

	***β*-SnMoO4**		***β*-SnWO4**		***α*-SnWO4**				***α*-SnMoO4**			
	a=b=c	E_{gap}	***a=b=c***	E_{gap}	***a***	***b***	***c***	E_{gap}	***a***	***b***	***c***	E_{gap}
**B3LYP**	7.26	3.94	7.16	4.78	5.75	10.42	5.68	2.65	5.81	10.42	5.74	1.80
**Diferences (%)**	**0**	**--**	**-1.78**	**78.36**	**2.31**	**-10.48**	**13.83**	**61.58**	**3.38**	**-10.48**	**15.03**	
**HSE06**	7.13	3.37	7.07	4.15	5.61	10.57	5.50	2.14	5.60	10.72	5.42	1.34
**Diferences (%)**	**-1.80**	**--**	**-3.01**	**55.22**	**-0.35**	**-9.19**	**10.22**	**30.48**	**-0.35**	**-7.99**	**8.62**	
**Experimental Reference**	7.26[19]	--]	7.29[18]	2.68[18]	5.62[18]	11.64[18]	4.99[18]	1.64[18]	5.62[18]	11.64[18]	4.99[18]	
**Theoretical Reference**	7.54[19]	3.74[19]	7.39[18]	3.45[18]	5.59[18]	11.69[18]	4.99[18]	1.65[18]				

Table S2. Optimized lattice parameters of the α and β structures of the $SnMo_{1-x}W_xO_4$ (x = 0, 0.25, 0.50, 0.75, and 1) solid solutions along with calculated EOS parameters and band gap values.

***α*-Phase**	**$SnWO_4$**	**$SnW_{0.75}Mo_{0.25}O_4$**	**$SnW_{0.50}Mo_{0.50}O_4$**	**$SnW_{0.25}Mo_{0.75}O_4$**	**$SnMoO_4$**
a (Å)	5.605	5.600	5.597	5.590	5.597
b (Å)	10.574	10.582	10.610	10.691	10.717
c (Å)	5.498	5.487	5.469	5.427	5.423
**V** (Å^3)	325.94	325.22	324.83	324.10	325.45
**B_0** (GPa)	69.47				57.60
**B'_0**	4.98				5.33
**Egap** (eV)	2.14	1.61	1.38	1.39	1.34
***β*-Phase**	**$SnWO_4$**	**$SnW_{0.75}Mo_{0.25}O_4$**	**$SnW_{0.50}Mo_{0.50}O_4$**	**$SnW_{0.25}Mo_{0.75}O_4$**	**$SnMoO_4$**
**a** (Å)	7.073	7.076	7.089	7.115	7.131
**b** (Å)	7.073	7.076	7.102	7.115	7.131
**c** (Å)	7.073	7.076	7.110	7.115	7.131
**V** (Å^3)	353.97	353.15	357.89	360.21	362.64
**B_0** (Gpa)	40.80				41.93
**B'_0**	7.22				5.65
**Egap** (*eV*)	4.15	3.23	3.22	3.29	3.37

Table S3. Optimized atomic positions in cartesian coordinates for the α and β structures of $SnMo_{1-x}W_xO_4$ (x = 0, 0.25, 0.50, 0.75, and 1) solid solutions.

α-$SnMoO_4$

	x	y	z
Sn	1.399582984	0	1.247593806
Mo	-1.534394528	2.678618845	1.356430651
O1	2.394477758	3.525203975	-0.238585816
O2	0.556428717	4.157763919	-2.220641823

β-$SnMoO_4$

	x	y	z
Sn	-1.003446067	-1.003446067	-1.003446067
Mo	1.149559561	1.149559561	1.149559561
O1	2.188630691	2.188630691	2.188630691
O2	-0.912633084	-1.548230497	-3.171277541

α-$SnWO_4$

	x	y	z
Sn	1.401422972	0	1.24958438
Mo	-1.467965496	2.64368161	1.37461835
O1	2.393844724	3.533405043	-3.237345244
O2	0.53075447	4.013502969	-2.271782778

β-$SnWO_4$

	x	y	z
Sn	-9.94778367	-9.94778367	-9.94778367
W	1.144352421	1.144352421	1.144352421
O1	2.160309292	2.160309292	2.160309292
O2	-0.866551666	-1.57667516	-3.162159637

α-$SnMo_{0.25}W_{0.75}O_4$ (Space Group P2 No. 3)

	x	y	z
Sn1	1.411365044	-0.009461718	1.24622833
Sn2	-1.360922495	-0.004969603	-1.266770514
Mo	-1.544603227	2.646528994	1.374374678
W1	-1.354785467	-2.644540869	1.369274251
W2	1.422532454	-2.646528994	-1.374374678
W3	1.322900756	2.644540869	-1.369274251
O1	2.38997326	3.450917162	-0.254887181
O2	0.378531185	-3.600737606	-0.349725814
O3	-2.405211745	-3.596060307	0.361257395
O4	-0.417989479	3.489510134	0.263175051
O5	0.582391448	4.095624961	-2.189278308
O6	2.271195233	-3.951000604	-2.298527845
O7	-0.513777004	-3.958881849	2.31085694
O8	-2.258577704	4.124576785	2.249662953

β-$SnMo_{0.25}W_{0.75}O_4$ (Space Group R3 No. 146)

	x	y	z
Sn1	-0.950701635	-0.950701635	-0.950701635
Sn2	-2.674891234	0.986164022	2.455227668
Mo	1.015864361	1.015864361	1.015864361
W	2.628494829	-1.230997013	-2.456429128
O1	2.05621497	2.05621497	2.05621497
O2	1.279582582	-2.127117118	-1.79725186
O3	-1.092577021	-1.138212141	-3.12420392
O4	-2.663018489	2.098537984	0.416285712
O5	3.000420645	-2.019540755	3.053572356
O6	0.760667603	1.991388647	-0.467481067

α-$SnMo_{0.50}W_{0.50}O_4$ (Space Group $P22_12$ No. 17)

	x	y	z
Sn	1.338186162	0.009252515	1.26578916
W	-1.375429976	2.645332992	1.368851448
Mo	-1.237179005	-2.645332992	1.368851448
O1	2.441041861	3.676570963	-0.392266662
O2	0.421084163	-3.357954437	-0.1608942
O3	0.522106459	3.876660645	-2.341445597
O4	2.165628879	-4.225329353	-2.14349098

β-$SnMo_{0.50}W_{0.50}O_4$ (Space Group $P2_1$ No. 4)

	x	y	z
Sn1	-1.040638566	-0.974515755	-1.058865785
Sn2	-2.515860562	1.034590838	2.540351673
W	1.195007168	1.15083618	1.207337642
Mo	2.380383106	-1.132976283	-2.40416327
O1	2.175474534	2.200632052	2.225504476
O2	1.320012454	-2.148126698	-1.363793047
O3	-1.033083779	-1.630816178	-3.203200221
O4	-2.73558359	1.503108322	0.364812804
O5	-3.220862121	-0.888947224	-1.5757918
O6	-1.545721475	-3.173391761	-0.818995549
O7	-0.363636868	0.892990935	1.954520669
O8	-1.940039958	3.166615571	2.530034037

α-$SnMo_{0.75}W_{0.25}O_4$ (Space Group P2 No. 3)

	x	y	z
Sn1	1.386729148	-0.010721913	1.20380533
Sn2	-1.330216009	-0.00878396	-1.21152826
Mo1	-1.614766612	2.762084144	1.297267933
Mo2	-1.175014293	-2.771375438	1.311857381
Mo3	1.594217314	-2.762084144	-1.297267933
W	1.134732961	2.771375438	-1.311857381
O1	2.232479433	3.387116357	-0.150300004
O2	0.440156075	-3.573061264	-0.170341686
O3	-2.324678318	-3.564920828	0.178825789
O4	-0.463682114	3.408608424	0.067514862
O5	0.596509996	4.410801671	-2.080114067
O18	2.23983	-4.28006	-2.15527
O7	-0.543048409	-4.312568553	2.159898062
O8	-2.203667539	4.351360195	2.060498127

β-$SnMo_{0.25}W_{0.75}O_4$ (Space Group R3 No. 146)

	x	y	z
Sn1	-1.004450214	-0.947113391	-1.034619067
Sn2	0.979198018	2.551218615	-2.551218615
Mo	1.130818696	1.105603568	1.156514061
W	-1.144259042	-2.386157591	2.386157591
O1	2.148353118	2.161625595	2.199494574
O2	-2.160725042	-1.369691591	1.369691591
O3	-0.988925574	-1.567663444	-3.170712153
O4	-2.662270561	1.542019658	0.360138525
O5	2.772095823	-2.073808399	3.177807262
O6	0.88662755	2.001566818	-0.369208109

Table S4. Elongation (λ) and angular variance (σ) distortion parameters of MoO_4 and WO_4 tetrahedra in the β and α phases.

	**β-$SnMoO_4$**	**β-$SnWO_4$**	**α-$SnMoO_4$**	**α-$SnWO_4$**
λ	1.0015	1.0001	1.047	1.028
Σ	6.14	0.36	186.54	103.81

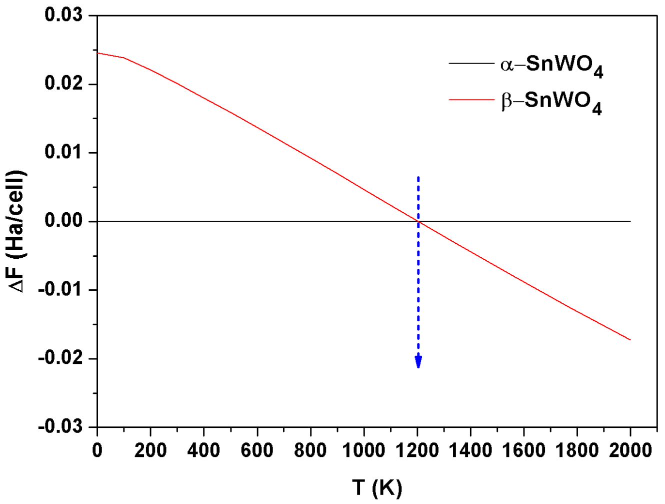

Figure S1: Variation with temperature of the Gibbs energy of β-$SnWO_4$ .with respect to α-$SnWO_4$.

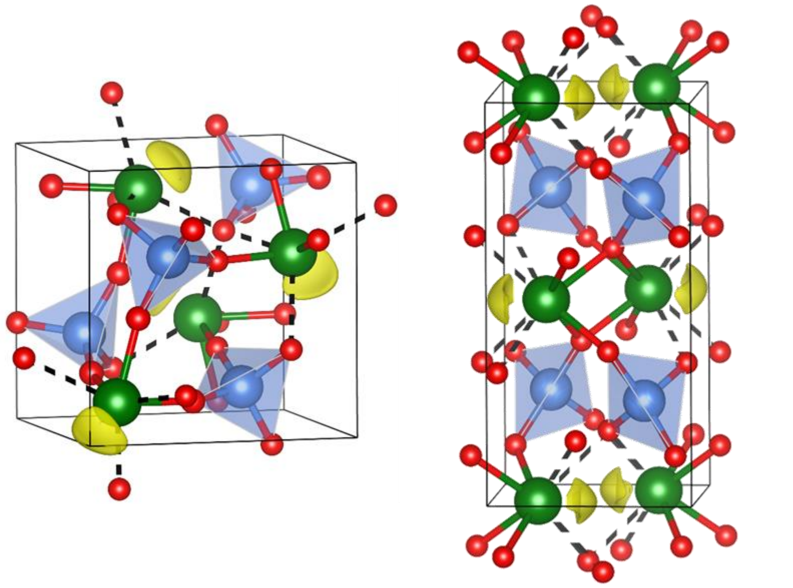

Figure S2. β and α structures highlighting the lone electron pair regions (yellow) associated with the SnO_3E and SnO_4E units. Sn-O bonds within these units are depicted as green-red bicolor cylinders, whereas the remaining Sn-O bonds of the SnO_6 (β-phase) and SnO_8 (α-phase) polyhedral are shown as dashed black lines. Blue transition metal polyhedral are highlighted. Green, and red balls stand for Sn and O atoms, respectively.

1 Dovesi, R.; Erba, A.; Orlando, R.; Zicovich-Wilson, C. M.; Civalleri, B.; Maschio, L.; Rerat, M.; Casassa, S.; Baima, J.; Salustro and S.; Kirtman. B. Quantum-Mechanical Condensed Matter Simulations with CRYSTAL. *Comput Mol Sci*. **2018**, *8*, e1360:1-36.

2 Dovesi,R.; Saunders, V. R.; Roetti, C.; Orlando, R.; Zicovich-Wilson, C. M.; Pascale, F.; Civalleri, B.; Doll, K.; Harrison, N. M.; Bush, I. J.; D'Arco, P.; Llunell, M.; Causà, M.; Noël, Y.; Maschio, L.; Erba, A.; Rerat, M.; and Casassa S. CRYSTAL17 User's Manual (University of Torino, Torino (**2017)**.

3 Heyd, J.; Scuseria, G. E. and Ernzerhof, M; Hybrid Functionals based on a Screened Coulomb Potential *J. Chem. Phys.* **2003**, *118*, 8207-8215.

4 Krukau, A. V.; Vydrov, O. A.; Izmaylov, A. F.; and Scuseria, G. E. Influence of the Exchange Screening Parameter on the Performance of Screened Hybrid Functionals. *J. Chem. Phys.* **2006** *125*, 224106:1-5.

5 Becke. A.D. Density-Functional Thermochemistry. III. The Role of Exact Exchange. *J. Chem. Phys.* **1993**, *98*, 5648-5652.

6 Corà, F.; Patel, A.; Harrison, N. M.; Dovesi, R.; Catlow, C. R. A. An Ab-initio Hartree-Fock Study of the Cubic and Tetragonal Phases of Bulk Tungsten Trioxide *J. Am. Chem. Soc.* **1996**, *118*, 12174-12182.

7 Gomes, E.O.; Fabris, G. S. L.; Ferrer, M.M.; Motta, F.V.; Bomio, M. R. D.; Andres, J.; Longo, E.; Sambrano, J. R. Computational Procedure to an Accurate DFT Simulation to Solid State Systems. *Comput. Mater. Sci.* **2019**, *170*, 109176:1-10.

8 Sophia, G.; Baranek, P.; Sarrazin, C.; Rerat, M.; Dovesi, R. Systematic Influence of Atomic Substitution on the Phase Diagram of ABO_3 Ferroelectric Perovskites. **2014** (Unpublished) https://www.crystal.unito.it/Basis_Sets/tin.html. Visited on 01/03/2022.

9 Corno, M.; Busco, C.; Civalleri, B.; Ugliengo, P. Periodic Ab-Initio Study of Structural and Vibrational Features of Hexagonal Hydroxyapatite $Ca_{10}(PO_4)_6(OH)_2$ *Phys. Chem. Chem. Phys*. 2006, *8*, 2464-2472.

10 Birch, F. Finite Elastic Strain of Cubic Crystals. *Phys. Rev.* **1947**, *71*, 809-824.

11 Murnaghan, F.D. The Compressibility of Media under Extreme Pressures. *Proc. Natl. Acad. Sci. USA* **1944**, *30*, 244-247.

12 Kresse, G.; Furthmuller, J. Efficient Iterative Schemes for Ab Initio Total-Energy Calculations Using a Plane-Wave Basis Set. *Phys. Rev. B* **1996**, *54*, 11169- 11186.

13 Kresse, G.; Furthmüller, J. Furthmuller, Efficiency of Ab-Initio Total Energy Calculations for Metals and Semiconductors Using a Plane-Wave Basis Set. *Comput. Mater. Sci.* **1996**, *6*, 15-50.

14 Perdew, J. P.; Ruzsinszky, A.; Csonka, G. I.; Vydrov, O. A.; Scuseria, G. E.; Constantin, L. A.; Zhou, X.; Burke, K. Restoring the Density-Gradient Expansion for Exchange in Solids and Surfaces. *Phys. Rev. Lett*. **2008**, *100*, 136406:1-4.

15 Kresse, I. G. and Joubert, D. From Ultrasoft Pseudopotentials to the Projector Augmented-Wave Method. *Phys. Rev. B* **1999**, *59*, 1758-1775.

16 Deringer, V. L.; Tchougr ́eeff, A. L.; Dronskowski, R. Crystal Orbital Hamilton Population (COHP) Analysis As Projected from Plane-Wave Basis Sets. *J. Phys. Chem. A* **2011**, *115*, 5461-5466.

17 Maintz, S.; Deringer, V. L.; Tchougréeff, A. L.; Dronskowski, R. LOBSTER: A Tool to Extract Chemical Bonding from Plane-Wave based DFT. *J. Comput. Chem.* **2016**, *37*, 1030-1035.

18 Kuzmin, A.; Anspoks, A.; Kalinko, A.; Timoshenko, J.; Kalendarev, R. "External Pressure and Composition Effects on the Atomic and Electronic Structure of $SnWO_4$, *Sol. Energy Mater. Sol. Cells* **2015**, *143*, 627–634.

19 Hayashi, H.; Katayama, S.;Komura, T.; Hinuma, Y.; Yokoyama, T.; Mibu, K.; Oba, F.; Tanaka, I. "Discovery of a Novel Sn(II)-Based Oxide β-$SnMoO_4$ for Daylight-Driven Photocatalysis," *Adv. Sci.* 4, **2017**,*1*, 1–8.
