# Supplementary material for: A Chemical-Pressure-Induced Phase Transition Controlled by Lone Electron Pair Activity
Source: J Phys Chem Lett. 2022 Oct 17;13(42):9883–8. doi: 10.1021/acs.jpclett.2c02582 (PMC9619963; doi:10.1021/acs.jpclett.2c02582)
Supplement: Supplementary file 2 — jz2c02582_si_002.pdf [file jz2c02582_si_002.pdf]

jz-2022-02582h.R1

Name: Peer Review Information for "A Chemical Pressure-Induced Phase Transition Controlled by Lone Electron Pair Activity"

First Round of Reviewer Comments

Reviewer: 1

Comments to the Author

This letter reports an interesting chemical pressure driven alfa-beta phase transition in Sn(Mo,W)O4 compounds, and give a clear understanding of the correlations among physical pressure, lone pair activity, coordination environment, and phase transition. These are important indications to understand the unusual properties in Pb, Sn, Bi-based materials in solid state chemistry community. I have two comments/suggestions:

1, The similar ionic radius (i.e., 0.59 A and 0.60 A for Mo6+ and W6+ in octahedral environment) indicates a dominated contribution of element properties, i.e. electronegativity as the author discussed. As mentioned in Ref 5, chemical pressure controls material properties not by size effect alone, but also by some indirect effect of the size effect. More discussion on this point would increase the rationality of the logic.

2. While the physical significance is important, If possible, I suggest to experimentally verify the predicted Mo-W cross substitution modulated phase transition.

Reviewer: 2

Comments to the Author

The structural preferences of the SnMoO4 and SnWO4 phases based on external pressure and temperature variables, as well as the SnMo1-xWxO4 solid solution are studied using the concept of chemical pressure. Although Mo and W share similar atomic size, the SnMoO4 and SnWO4 phases prefer at ambient conditions, respectively, the P213 (beta) and Pnna α(alpha) phases and undergo transitions to the other at, respectively, high pressure or high temperature. By considering as well the Mo/W fraction at which SnMo1-xWxO4 undergoes a structural transformation, the Mo/W fraction, as a chemical pressure, can be associated with the physical pressure at which the SnMoO4 and SnWO4 end members would have the same energy difference between the two phases. This ultimately links the temperature and pressure environmental variables.

Then, the presence of the strong distorter Mo^{6+} cation, more electronegative than the weak distorter W^{6+}, allows for stronger Mo-O bonds (with O atoms also bound to Sn as bridging atoms) and thus stronger hybridization between the Sn 5s and 5p states. The authors suggest that the increased stereoactivity of the Sn lone pair would prefer the more rigid SnO_3E units found in the less symmetric P213 β phase, and would give cause for transition to the denser Pnna α phase under pressure.

A few minor points:

- In Fig 4 should the Xw=0 and Xw=0.25 positions be flipped?

- Some more (or more explicit) discussion of how high temperature favors the beta phase for $SnWO_6$ would be helpful

- It would be nice to get a sense of the significance of the results in a broad context. Are they only relevant for the $SnMo_{1-x}W_xO_4$ (x = 0-1) compounds discussed herein? Could they be generally applicable? What can researchers learn from these results in designing a material with a particular structure and for a given function?

- the manuscript is a bit long for a letter

Reviewer: 3

Comments to the Author

Overall this paper is suitable for JPC Letters. I did get a little confused with respect to the alpha and beta phases for $SnMoO_4$ and $SnWO_4$. Figure 1 helps. But if I understand correctly, it is not possible to transform from b-$SnWO_4$ ---> b-$SnMoO_4$, but you can transform a-$SnWO_4$ ---> b-$SnMoO_4$. Is that correct? It seems a b(W) --> b(Mo) transformation should be possible at elevated temperatures, but upon cooling b(Mo) --> b(W). Am I understanding this correctly? If so, this could be explained better in the paper. Minor comment - associated errors should be given for all the lattice parameters on page 6.

Author's Response to Peer Review Comments:

Dear Prof. Editor,

Thank you very much for your email informing us on the decision on our manuscript and for sending us the corresponding referee's reports. We have carefully read the reports and we are glad to see that the reviewers have taken their time to send us a comprehensive assessment of our manuscript. We are grateful for their frank account of our study. Even though the Reviewer 2 was not favorable to recommend our manuscript in JPCL, his/her report does not introduce any fundamental criticism against the conclusions of our study and only raises doubt on the suitability of JPCL as the appropriate journal to publish our manuscript. After addressing the comments of the reviewers, we hope that the amended manuscript will be suitable to be published in the JPCL. We have also revised the references format and other non-scientific changes suggested in your letter. A detailed point-by-point response to the reviewers is provided below highlighting in green the new sentences added to the amended manuscript.

Best Regards,

J. Manuel Recio
Prof. of Chemical Physics
University of Oviedo (Spain)
jmrecio@uniovi.es

Alvaro Lobato, PhD
Assistant Professor
Complutense University (Spain)
a.lobato@ucm.es

Reviewer: 1

Comment1. The similar ionic radius (i.e., 0.59 A and 0.60 A for Mo^{6+} and W^{6+} in octahedral environment) indicates a dominated contribution of element properties, i.e. electronegativity as the author discussed. As mentioned in Ref 5, chemical pressure controls material properties not by size effect alone, but also by some indirect effect of the size effect. More discussion on this point would increase the rationality of the logic.

Answer1. The point raised by the reviewer is one of the key messages we wanted to highlight in our manuscript (see for example the first paragraph of the introduction). It seems that we have not been clear enough in this regard. As the reviewer ask us to emphasize on this point, we have cited again Ref. 5 when this point is addressed in the introduction and, more importantly, we have included new sentences in the discussion and in the conclusion paragraph of the manuscript about this specific point. We have also added a reference where the activity of the lone pair has been discussed on the grounds of the DFT-chemical pressure analysis (Ref 25).

The new sentences that we have introduced are:

Page 10, second paragraph. The activity of the lone pair is known to be dependent on the electronegativity of the ligand.[25]

Page 10, second paragraph. As emphasized by Lin et al.,[5] chemical pressure induces modifications in the material behaviour caused not only by size effects but also due to new chemical interactions emerging from changes in the electronic structure.

Page 13, first paragraph. This study highlights the usually overlooked role played by the electronic structure of the guest atom when discussing chemical pressure effects. Since size effects are not meaningful in our system due to similar ionic radii of the two 3d cations, electronic structure modifications are here the main responsible to control the thermodynamic stability of particular compositions of the $SnMo_{(1-x)}W_xO_4$ solid solution by appropriate chemical doping.

Comment2. While the physical significance is important, If possible, I suggest to experimentally verify the predicted Mo-W cross substitution modulated phase transition.

Answer2. This suggestion is totally pertinent, although is far from the scope of our study. In fact, we were looking in vain for experimental data reporting the alloy composition at which the alpha-beta transition occurs. In this sense, the predicted x_W=0.43 value could be seen as a challenge and as a guide for new experiments in this alloy. A new sentence has been added in the conclusion paragraph of the modified version of the manuscript.

Page 13, first paragraph. To the best of our knowledge, this is the first time a phase transition induced by the modification of the lone pair activity upon chemical substitution is reported. We believe that experimental proof of such transition would constitute a plausible challenge for being detected in the laboratory.

Reviewer:2
Comment1. In Fig 4 should the Xw=0 and Xw=0.25 positions be flipped?

Answer1. These values are correct. It could be in the other way around as the reviewer probably expect, but we found that the solid solution does not follow an "ideal" trend. This means that one particular pressure (between -2 and -4 GPa approximately) can be found for two different compositions. Starting with x_W=0 the pressure decreases as x_W increases up to 0.25, and then pressure increases when x_W increases up to x_W=1. In energetic terms, this means that the solution starts to show preference for the alpha structure when x_W values are higher than 0.25.

Comment2. Some more (or more explicit) discussion of how high temperature favors the beta phase for SnWO6 would be helpful

Answer2. The reviewer has detected that we have not been explicit in explaining the reasons that favors the beta phase for $SnWO_4$ at high-temperature. However, we have provided in the manuscript the reasons why the beta phase is preferred at negative pressures for $SnWO_4$: 'Similarly, the preference for the most rigid and more active lone pair of the β phase in the case of the W compound compensates the decrease in the coordination number when transforming from the α phase'. We assume that these arguments can be transferred to the high temperature regime since volumes are the same at the transition temperature and at the transition negative pressure. This last sentence has been added to the previous paragraph of the conclusion paragraph in the amended version of the manuscript.

Comment3. It would be nice to get a sense of the significance of the results in a broad context. Are they only relevant for the $SnMo_{1-x}W_xO_4$ (x = 0-1) compounds discussed herein? Could they be generally applicable? What can researchers learn from these results in designing a material with a particular structure and for a given function?

Answer3. We have tried to show that the overall message is not specific to this alloy and can be generalized to other systems. The fact that chemical pressure can be associated with physical pressure opens the possibility of replacing one of the two kind of "pressures" by the other when carrying experiments. We are not the first ones saying this. See for example Refs 1 and 5 of the manuscript, but, as far as we know, we illustrate for the first time how lone pair activity and not a pure size effect is the responsible to induce new properties by means of chemical pressure. As also suggested by the reviewer 1, this point has been emphasized in the amended version of the manuscript (See above in the answer to reviewer 1). Moreover, in the conclusion paragraph we have explicitly addressed the potential implications of our work in other properties and materials.

The new text included is:

Page 13, first paragraph. Extensions to other crystal families containing ns^2 lone pair cations (i.e. Pb, Se, Bi, etc.) and covering interesting phenomena such as multiferroic behaviour, low thermal conductivity or non-linear optics are worth to be explored following our approach since lone pair activity might be used to effectively design new functional materials.

Comment4. the manuscript is a bit long for a letter

Answer4. We were also worried about this point and tried to condense as much as possible our manuscript although we agree that it is not a short one. However, we have seen other contributions in JPCL that are similar in length to ours.

Reviewer: 3

Comment1. Overall this paper is suitable for JPC Letters. I did get a little confused with respect to the alpha and beta phases for $SnMoO_4$ and $SnWO_4$. Figure 1 helps. But if I understand correctly, it is not possible to transform from b-$SnWO_4$ ---> b-$SnMoO_4$, but you can transform a-$SnWO_4$ ---> b-$SnMoO_4$. Is that correct? It seems a b(W) --> b(Mo) transformation should be possible at elevated temperatures, but upon cooling b(Mo) --

> b(W). Am I understanding this correctly? If so, this could be explained better in the paper.

Answer1. To obtain β-$SnMoO_4$ from β-$SnWO_4$, first β-$SnWO_4$ has to be transformed into a-$SnWO_4$ by decreasing temperature. Then, by replacing W with Mo, α-$SnWO_4$ converts into β-$SnMoO_4$. The potential transformations are the ones that connect structures and compounds with arrows in Figure 1.

Comment2. Minor comment - associated errors should be given for all the lattice parameters on page 6.

Answer2. Determining errors from solid state calculations is a difficult task because they depend on different parameters such as the functional used, the cut-offs, k-point mesh, etc. With the methodology and convergence criteria used in our calculations, the accuracy on the lattice parameters is of the order of 10^{-3} Å. As usually reported in computational studies, errors are not included in the tables although a sentence warning the accuracy of our data is included in Page 6 of the amended version of the manuscript.
